# Pathogen-specific predicting factors of childhood diarrhoea and their seasonality: evaluation from Rohingya refugees and host population in Cox’s Bazar, Bangladesh

**DOI:** 10.7189/jogh.16.04024

**Published:** 2026-01-23

**Authors:** Nusrat Jahan Shaly, Sharika Nuzhat, Monira Sarmin, Nasif Hossain, Nafisa Mariam, Shams E Tabriz Bhuiyan, Md Ali Amin Nabin, Md Tariqujjaman, Md Ahshanul Haque, Dilruba Ahmed, A S G Faruque, Tahmeed Ahmed, Mohammod Jobayer Chisti

**Affiliations:** 1 International Centre for Diarrhoeal Disease Research (icddr,b), Bangladesh, Dhaka, Bangladesh; 2 Division of Infectious Diseases and International Health, University of Virginia School of Medicine, Charlottesville, Virginia, USA; 3 Environmental Institute, University of Virginia, Charlottesville, Virginia, USA

## Abstract

**Background:**

Bangladesh observed a sudden massive influx of Rohingya refugees in August 2017. This large migrant population relative to a smaller host community placed a burden and threat on the public health sector. Due to the lack of pathogen-specific predicting factors and the influence of seasonal variation on childhood diarrhoeal pathogens in a densely populated area, we aimed to explore the same among Rohingya refugees and the host population.

**Methods:**

We collected data from under-five children of Rohingya refugees and hosts between 2018 and 2023 from the Diarrhea Treatment Center (DTC)-based surveillance system that served our study population. We collected and tested stool samples to detect enteric pathogens. We performed a multiple logistic regression analysis to identify factors associated with individual pathogens.

**Results:**

Out of 3534 children, 1479 (41.9%) were Rohingya refugees, and 2055 (58.1%) were host children who visited DTCs. Bacterial pathogens were identified in 15% (n/N = 533/3534) of children, and rotavirus in 58% (n/N = 1492/2564). We found higher odds of *Vibrio cholerae* (adjusted odds ratio (aOR) = 2.12; 95% confidence interval (CI) = 1.21–3.74), non-typhoidal *Salmonella* (NTS) (aOR = 4.45; 95% CI = 2.04–9.68), and lower odds of rotavirus infection (aOR = 0.72; 95% CI = 0.59–0.89) during the wet season compared to the cold season. Lack of handwashing with soap before feeding the child increased the risk of *Aeromonas* infection (aOR = 1.85; 95% CI = 1.21–2.81). Drinking tube well water lowers the risk of *Vibrio cholerae* (95% CI = 0.24–0.71), rotavirus (95% CI = 0.57–0.86), and *Aeromonas* (95% CI = 0.36–0.75) infection. We found that the recent intake of vitamin A was a protective factor for *Vibrio cholerae* (95% CI = 0.26–0.76), *Aeromonas* (95% CI = 0.44–0.89), and NTS (95% CI = 0.12–0.56) enteric infections.

**Conclusions:**

Our results underscore the necessity of reinforcing routine diarrhoea surveillance for early detection of epidemics, vitamin A supplementation for children under five, and health education to prevent diarrhoea in vulnerable areas such as refugee camps.

Diarrhoea is the second leading cause of under-five (U5) deaths caused by bacterial, viral, and parasitic pathogens [1–3]. Globally, in 2021, diarrhoea accounted for 8.7% of deaths among U5 children, amounting to 5.10 million deaths [4]. Due to advancements in diarrhoea treatment with oral rehydration solution (ORS) and zinc, as well as improved sanitation and care-seeking behaviour, worldwide, the number of U5 deaths declined by 2.5% from 2000 to 2021. Still, there were 3.3 deaths per thousand live births [4,5]. The burden of diarrheal diseases and deaths was partly borne by the children living in the different settlements in low- and middle-income countries due to poor living conditions. An informal settlement in Kenya found *E coli* to be the predominant enteric pathogen associated with diarrhoea in U5 children [6]. Data from the United Nations High Commissioner for Refugees (UNHCR) found that cholera was one of the major causes of epidemics in the refugee camps. Besides *Vibrio cholerae*, *Salmonella,* and *Shigella* were also reported to cause epidemics in the refugee camps [7–9]. A cholera outbreak was reported from Cox’s Bazar, Bangladesh, among the Rohingya refugees, and about 47% of the cholera cases were found in U5 children [10]. It was perceived that mortality and severity of diarrhoea in children depend on the type of pathogen and risk factors. Moreover, geographical location and seasonal variation influence the variance in diarrhoeal pathogens [11].

Globally, there were 258.2 million episodes of rotavirus diarrhoea in U5 children and 20.3 deaths per 100 000 in 2016 [12]. Being the predominant causative organism for diarrhoea in U5 children, 54% of the total diarrhoea admissions in Bangladesh were due to rotavirus [1,13]. A similar status was reported in Myanmar, where 42–56% of diarrhoea among U5 children was due to rotavirus [1]. Apart from the rotavirus, cholera remains a significant pathogen for diarrhoea in children, especially in low- and middle-income countries [14]. Due to the Ganges Delta, Bangladesh is a hub for cholera, with a seasonal peak in both the pre- and post-monsoon seasons [14]. Similar to Bangladesh, Myanmar is also a cholera-endemic country with a case fatality rate of 1.4% [2].

Besides rotavirus and *Vibrio cholerae, Aeromonas* species, an important pathogen for diarrhoea in children, were found predominantly in developing countries' seafood, saline water, soil, and animal faeces [15,16]. The Global Enteric Multicenter Study reported a 22.2% prevalence of moderate-to-severe diarrhoea in children by *Aeromonas* in Bangladesh and Pakistan [17]. In sub-Saharan Africa and South Asia, non-typhoidal *Salmonella* (NTS) infection was often associated with growth faltering among U5 children [18].

Waterborne diseases, like diarrhoea, have a direct correlation with overpopulation due to poor quality of life, scarcity of safe water, lack of safe sanitation and hygiene practices, insecure waste and child faeces disposal, and living in poorly built houses [19]. Overcrowding is more prominent in refugee camps or settlements, which are prone to outbreaks of infectious diseases, including diarrhoea [20].

Rohingya Refugees, a group of people who fled from Myanmar, are regarded as one of the most victimised people in the world. In late August 2017, there was a mass influx of Rohingya refugees, and they took shelter in the coastal part of Bangladesh. According to the joint report of government of Bangladesh and UNHCR on 30 November 2023, about one million Rohingya refugees reside in 33 extremely congested camps in Ukhiya and Teknaf Upazilas of the Cox’s Bazar District, as well as on the island of Bhasan Char [21]. With a total Rohingya refugee population of 938 280, Cox’s Bazar has become the largest refugee camp in the world. The massive influx of such a large number of Rohingya refugees over six years has had direct and indirect effects on the more than half a million (n = 541 021) Bangladeshi host communities who live near these settlements [22]. The densely populated living conditions in the settlements and the proximity of the unvaccinated host population against cholera, including the lack of basic amenities, made the children vulnerable to a cholera outbreak [10].

The enteric pathogens associated with diarrhoea among Rohingya refugees and neighbouring host children have great importance not only for their clinical features and severity but also for improving public health policy, reinforcement of vaccination programs, and health education for these vulnerable populations to prevent diarrhoea and related adverse events in young children. We aimed to identify factors (demographic, care-seeking behaviour, clinical, nutritional status, and seasonality) associated with enteric pathogens in childhood diarrhoea among the study children.

## METHODS

### Study population and study site

The study population was U5 children (0–59 months old) of any sex attending the Diarrhea Treatment Center (DTC) for diarrhoea between 22 April 2018 and 30 April 2023. There were 3534 children enrolled in the surveillance during the study period. After we excluded children with mixed pathogens or few pathogens, the total sample size was 3298. DTCs were located within the Rohingya refugee camps. The neighbouring host, who had U5 children, also sought healthcare from the International Centre for Diarrhoeal Disease Research, Bangladesh (icddr,b)-run DTCs and was included in this study. Due to their geographical, social, and epidemiological similarities, they were susceptible to similar enteric pathogens. Details about the DTCs and surveillance were described elsewhere [23]. In the DTCs, diarrhoeal children were managed with either ORS or intravenous fluid, oral zinc, and antibiotics for invasive diarrhoea or cholera. There were also facilities for treating malnutrition, pneumonia, and other common paediatric diseases.

### Study design

We did a secondary data analysis of previously collected DTCs surveillance data. Data were collected once during enrolment, and participants were not followed up. We compared children who had pathogens with those who had no pathogens in their stool. Henceforth, we used a case-control study design. Children who had identified only with *Vibrio cholerae,* rotavirus, NTS, and *Aeromonas* spp. were considered cases, while those with no pathogens in their stool were considered controls.

### Operational definition

Our outcome variables were the presence or absence of enteric pathogens, namely *Vibrio cholerae*, *Aeromonas* spp., NTS, and rotavirus in U5 children with diarrhoea attending DTCs for treatment. There was a total of seven *Shigella* spp. and four *Plesiomonas* identified from the study children. Due to this small case number, we did not include *Shigella* spp. and *Plesiomonas* in our outcome variables. We aimed to identify individual pathogen-specific predicting factors; thus, we had excluded mixed pathogens from the analysis. WHO defined diarrhoea as the passage of abnormally loose, watery stools, occurring ≥3 times in 24 hours [24].

We selected explanatory variables based on the literature search [2,18,25,26] and biological plausibility with the outcome variables. Variables described sociodemographic characteristics, breastfeeding status of the study children, water, sanitation, and hygiene (WASH) practices, healthcare-seeking behaviour, vaccination status, nutritional status, seasonal variation [27,28], and clinical characteristics associated with enteric pathogens (Table S1 in the **Online Supplementary Document**).

### Collection and testing of stool samples

At least 3 g of a single stool sample was collected from each patient enrolled in the surveillance. Immediately, a rapid dipstick test was performed for *Vibrio cholerae* using a rapid diagnostic test (RDT) kit [10,23]. Stool specimens of RDT-positive patients were then inoculated into the Cary-Blair Transport Medium to facilitate the growth of the organism and transported overnight to the central clinical microbiology and immunology laboratory of icddr,b Mohakhali, Dhaka, for culture confirmation and antibiotic susceptibility test. RDT-negative samples were sent once or twice weekly to Dhaka for microbial culture [10,29,30]. *Vibrio cholerae* and *Aeromonas* spp. were identified using Taurocholate Tellurite Gelatin Agar media in the clinical microbiology laboratory of icddr,b in Dhaka.

MacConkey and S-S agar (Salmonella-Shigella agar) were used to identify *Shigella* spp. and NTS. Rotavirus detection was performed using an enzyme immunoassay according to the manufacturer’s instructions (ProspectTM, Oxoid Diagnostics Ltd, UK).

### Data analysis

We used STATA, version 15.0 (Stata Corp LLC, College Station, Texas, USA) for all analyses. We expressed categorical variables as frequency (percentage) and median (interquartile range) for asymmetric continuous variables. We performed χ^2^ and Fisher exact test for the estimation of probability and to assess the relationship between the independent and outcome variables. We used the Kruskal-Wallis test to assess changes in the medians of different variables across the outcome variables. Additionally, we performed a multiple logistic regression analysis to investigate factors associated with enteric pathogens among the study children. We adjusted for similar covariates independently for each pathogen. The multiple logistic regression model used variables with a *P*-value of <0.05 in the bivariate analysis or had biological plausibility or potential public health impact. A *P*-value of <0.05 was considered statistically significant, and we reported adjusted odds ratios with 95% confidence intervals in the multivariable logistic regression analysis. We used backward elimination to obtain the final model in the logistic regression analysis. We checked for multicollinearity to exclude the overfitting of the variables. The average variance inflation factor for *Vibrio cholerae* was 1.66, 1.72 for rotavirus, 1.65 for *Aeromonas* spp., and 1.64 for NTS, indicating that the independent variables are minimally correlated with one another. Lastly, we conducted a Hosmer-Lemeshow goodness-of-fit test for each pathogen’s final multivariable regression model to assess model fit.

## RESULTS

**Figure 1:**
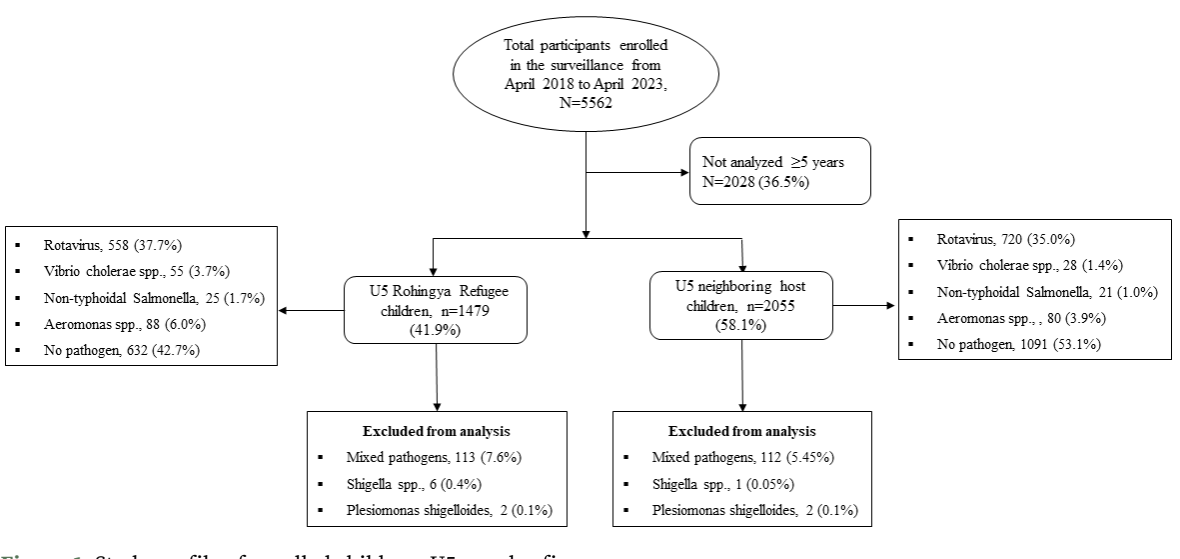
Study profile of enrolled children. U5 – under-five.

Of 3534 U5 children with diarrhoea, we detected bacterial enteric pathogens in 15.08% (n/N = 533/3534) and rotavirus in 58.2% (n/N = 2064/3534). The commonly isolated pathogens were rotavirus (37.7% *vs.* 35.0%), followed by *Aeromonas* spp. (6.0% *vs.* 3.9%), *Vibrio Cholerae* O1 and other *Vibrio* spp. (3.7% *vs.* 1.4%), NTS spp. (1.7% *vs.* 1.0%) among the Rohingya refugees and neighbouring host U5 children, respectively (**Figure 1**).

**Figure 2:**
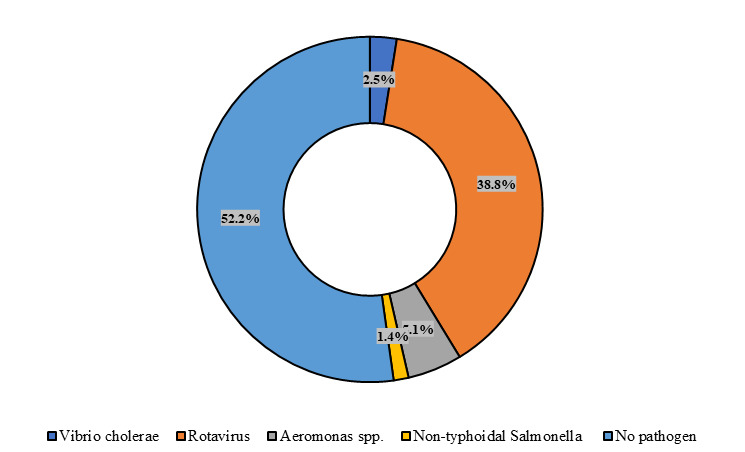
Percentage of enteric pathogens among study children.

There were 52.2% U5 children with no pathogens, whereas the prevalence of *Vibrio cholerae* was 2.5%, rotavirus 38.8%, *Aeromonas* spp. 5.1%, and NTS 1.4% as single enteric pathogens (**Figure 2**).

Regarding the sociodemographic factors, care-seeking behaviour, and clinical characteristics of U5 children with *Vibrio cholerae*, rotavirus, *Aeromonas* spp., and NTS showed that variables such as age, breastfeeding status, monthly family income, source of main drinking water, toilet shared with other households, handwashing practices with soap before feeding the child, distance to the main water source, number of U5 children in the family, Rohingya refugee children, season, intake of zinc, antibiotics, ORS at home prior hospitalisation, vitamin A intake within the last six months, distance travelled from home to hospital, stunting, duration of diarrhoea, watery stool, dehydration status, requiring intravenous fluids at the hospital, and duration of hospitalisation had a significant difference among pathogen categories, while other variables remain comparable (**Table 1**).

**Table 1:** Sociodemographic, care-seeking behaviour, and clinical characteristics of U5 children with enteric pathogens*

	*Vibrio cholerae* (n = 83)	Rotavirus (n = 1278)	*Aeromonas* spp. (n = 168)	NTS (n = 46)	No pathogen (n = 1723)	*P*-value
**Sociodemographic characteristics**						
Female sex	39 (47.0)	510 (39.9)	68 (40.5)	24 (52.2)	716 (41.6)	0.350
Age in months, MD (IQR)	25 (14–33)	11 (9–15)	12 (8–18)	12 (8–18)	11 (8–16)	<0.001
Currently breastfeed	35 (42.2)	1162 (90.9)	136 (80.9)	36 (78.3)	1468 (85.2)	<0.001
Monthly family income <9K	52 (62.6)	609 (47.6)	88 (52.4)	25 (54.3)	766 (44.5)	<0.001
Illiterate parent	76 (91.6)	1040 (81.4)	138 (82.1)	40 (86.9)	1420 (82.4)	0.175
Source of the main drinking water						
*Public tap*	48 (57.8)	468 (36.6)	74 (44.0)	23 (50.0)	480 (27.9)	<0.001
*Tube well*	32 (38.5)	755 (59.1)	86 (51.2)	21 (45.6)	1121 (65.1)	
*Others*	3 (3.6)	55 (4.3)	8 (4.8)	2 (4.3)	122 (7.1)	
Treatment of drinking water	7 (8.4)	210 (16.4)	25 (14.9)	10 (21.7)	262 (15.2)	0.240
Used a toilet without a water seal	74 (89.2)	1099 (86.0)	155 (92.3)	41 (89.1)	1487 (86.3)	0.207
Shared toilet with other households	58 (69.9)	680 (53.2)	96 (57.1)	27 (58.7)	828 (48.2)	<0.001
Practised handwashing with soap and water	5 (6.0)	127 (9.9)	10 (5.9)	2 (4.3)	151 (8.8)	0.293†
Practised handwashing with soap before feeding the child	70 (84.3)	1081/1252 (86.3)	126/163 (77.3)	44 (95.6)	1417/1694 (83.6)	0.005
Practised handwashing with soap after cleaning the child	80 (96.4)	1245 (97.6)	162 (98.8)	46 (100.0)	1662 (96.8)	0.368†
Consumed outside food 72 hours before diarrhoea	12 (14.5)	99 (7.7)	21 (12.5)	3 (6.52)	142 (8.2)	0.081†
Floor material of the house (cemented/ceramic tiles)	28 (33.7)	490 (38.3)	57 (33.9)	18 (39.1)	685 (39.8)	0.501
Number of people living in the house, MD (IQR)	6.00 (4.0–8.0)	5.0 (4.0–7.0)	6.0 (4.0–8.0)	5.0 (4.0–6.0)	5.0 (4.0–7.0)	0.056
Number of U5 children in the house, MD (IQR)	2.0 (1.0–2.0)	1.0 (1.0–2.0)	2.0 (1.0–2.0)	1.0 (1.0–2.0)	1.0 (1.0–2.0)	0.048
Distance to the main source of water in ft, MD (IQR)	40 (20–100)	20 (10–50)	30 (15–60)	30 (15–50)	20 (10–50)	<0.001
Got measles vaccine 1st dose	72/79 (91.1)	797/949 (83.9)	105/122 (86.0)	28/33 (84.8)	1065/1234 (86.3)	0.352‡
Rohingya refugee children	55 (66.3)	558 (43.7)	88 (52.4)	25 (54.3)	632 (36.7)	<0.001
Season						
*Wet (July–October)*	37 (44.6)	411 (32.2)	54 (32.1)	23 (50.0)	425 (24.7)	<0.001
*Cold (November–February)*	38 (45.8)	759 (59.4)	82 (48.8)	11 (23.9)	979 (56.8)	
*Hot (March–June)*	8 (9.6)	108 (8.4)	32 (19.0)	12 (26.1)	319 (18.5)	
**Care-seeking behaviour**						
ORS intake before hospitalisation	66 (79.5)	1066 (83.4)	132 (78.6)	34 (73.9)	1307 (75.9)	<0.001
Took zinc before hospitalisation	7 (8.4)	237 (18.5)	19 (11.3)	8 (17.4)	295 (17.1)	0.037
Took oral antibiotics before hospitalisation	13 (15.7)	387 (30.2)	34 (20.2)	10 (21.7)	573 (33.2)	<0.001
Received vitamin A within the last 6 months	25 (30.5)	590/1192 (49.5)	62/159 (38.9)	9/46 (21.4)	792/1567 (50.5)	<0.001
Distance travelled from home to hospital in km, MD (IQR)	3.0 (1.0–6.0)	3.0 (2.0–6.0)	3.0 (1.0–5.0)	2.0 (1.0–4.0)	3.0 (1.0–7.0)	0.005
**Nutritional status**						
Underweight (WAZ<−2)	29 (34.9)	327 (25.6)	50 (29.8)	7 (15.2)	446 (25.9)	0.109
Wasting (WLZ<−2)	28 (33.7)	299 (23.5)	32 (19.1)	7 (15.2)	397 (23.2)	0.078
Stunting (LAZ<−2)	23 (27.7)	210 (16.5)	32 (19.3)	12 (26.1)	311 (18.2)	0.048
**Clinical characteristics**						
Duration of diarrhoea >1 days on admission	38 (45.8)	848 (66.3)	95 (56.5)	19 (41.3)	1074 (62.3)	<0.001
Presence of abdominal pain	25 (30.1)	331 (25.9)	44 (26.2)	6 (13.0)	469 (27.2)	0.235
Watery stool	72 (86.7)	1074 (84.0)	125 (74.4)	33 (71.7)	1352 (78.5)	<0.001
Presence of dehydration	39 (46.9)	277 (21.7)	27 (16.1)	6 (13.0)	311 (18.0)	<0.001
Required intravenous rehydration	28 (33.7)	34 (2.7)	5 (2.9)	2 (4.3)	40 (2.3)	<0.001†
Duration of hospital stay in hours, MD (IQR)	18 (6–25)	8 (5–21)	6 (4–11.5)	5 (4–10)	6 (4–17)	<0.001

Cold season – November–February, Hot season – March–June, LAZ – length-for-age Z-score, IQR – interquartile range, MD – median, NTS – non-typhoidal Salmonella, ORS – oral rehydration solution, U5 – under-five, WAZ – weight-for-age Z-score, Wet season – July–October, WLZ – weight-for-length Z-score

*Values are presented as n (%) unless specified otherwise. Children with mixed pathogens and those with *Shigella* spp. and *Plesiomonas shigelloides* in the stool were excluded from this analysis.

†Results are from a Fisher's exact test.

The logistic regression analysis found that children aged 0–11 months and 12–23 months had a significantly lower risk of *Vibrio cholerae* infection and a higher risk of rotavirus infection than children aged 24–59 months. Children who used to drink tube well water and received vitamin A within the last six months had a lower risk of developing *Vibrio cholerae,* rotavirus, *Aeromonas*, and NTS infection (*P* < 0.05). There was a higher risk of having *Vibrio cholerae*, NTS, and a lower risk of rotavirus enteric infection during the wet season (July–October) compared to the cold season (November–February). Not practising handwashing with soap before feeding the child increased the risk of *Aeromonas* identification, whereas the use of oral antibiotics before hospitalisation decreased the risk. Children with rotavirus diarrhoea more commonly presented with watery stools and had longer hospital stays than those with no pathogen in the stool. Intravenous rehydration was more frequently required for children with cholera after admission at DTCs compared to children with no pathogen. NTS-positive children commonly visit DTCs with a shorter duration of diarrhoea compared to children with no isolated enteric pathogen (**Table 2**).

**Table 2:** Factors associated with enteric infections among U5 children*

	*Vibrio cholerae*		Rotavirus		*Aeromonas* spp.		NTS	
	**OR (95% CI)**	***P*-value**	**OR (95%CI)**	***P*-value**	**aOR (95% CI)**	***P*-value**	**aOR (95% CI)**	***P*-value**
**Age in months**								
24–59	ref		ref		ref		ref	
0–11	0.04 (0.02–0.10)	<0.001	1.59 (1.07–2.36)	0.022	0.63 (0.38–1.02)	0.062	0.64 (0.25–1.63)	0.355
12–23	0.21 (0.12–0.37)	<0.001	1.80 (1.22–2.66)	0.003	0.75 (0.46–1.22)	0.249	1.14 (0.46–2.82)	0.778
**Sex**								
Male	ref		ref		ref		ref	
Female	1.20 (0.72–2.02)	0.479	0.99 (0.82–1.19)	0.954	0.85 (0.60–1.20)	0.365	1.47 (0.78–2.75)	0.231
**Currently breastfeed**								
No	ref		ref		ref		ref	
Yes			1.37 (0.98–1.92)	0.063				
**Main source of drinking water**								
Public tap	ref		ref		ref		ref	
Tube well	0.41 (0.24–0.71)	0.001	0.70 (0.57–0.86)	0.001	0.52 (0.36–0.75)	<0.001		
Others	0.29 (0.08–1.11)	0.072	0.44 (0.29–0.67)	<0.001	0.36 (0.15–0.87)	0.023		
**Hand wash with soap before feeding the child**								
Practised	ref		ref		ref		ref	
Not practised					1.85 (1.21–2.81)	0.004		
**Season**								
Cold season	ref		ref		ref		ref	
Wet	2.12 (1.21–3.74)	0.009	0.72 (0.59–0.89)	0.002			4.45 (2.04–9.68)	<0.001
Hot	1.09 (0.47–2.52)	0.832	0.27 (0.20–0.35)	<0.001			4.17 (1.77–9.85)	0.001
**ORS intake before hospitalisation**								
No	ref		ref		ref		ref	
Yes			1.84 (1.47–2.30)	<0.001				
**Oral antibiotic before hospitalisation**								
No	ref		ref		ref		ref	
Yes					0.57 (0.38–0.87)	0.009		
**Received vitamin A within last 6 months**								
No	ref		ref		ref		ref	
Yes	0.44 (0.26–0.76)	<0.01			0.63 (0.44–0.89)	0.010	0.26 (0.12–0.56)	0.001
**Duration of diarrhoea >1 days on admission**								
No	ref		ref		ref		ref	
Yes							0.44 (0.23–0.85)	0.014
**Watery stool**								
No	ref		ref		ref		ref	
Yes			1.89 (1.51–2.36)	<0.001				
**Required intravenous rehydration**								
No								
Yes	6.30 (3.04–13.04)	<0.001						
**Duration of hospital stay in days**	1.02 (1.00, 1.05)	0.012	1.03 (1.02–1.04)	<0.001				
**Hosmer-Lemeshow *P*-value**		0.9972		0.4117		0.7867		0.5580

aOR – adjusted odds ratio, CI – confidence interval, NTS – non-typhoidal *Salmonella*, OR – odds ratio, ORS – oral rehydration solution, U5 – under-five

*The final multiple logistic regression model was adjusted for each pathogen independently with no pathogen by similar variables; participant's age, sex, family monthly income, breastfeeding status, parental education, number of children under the age of 5 in the house, handwashing practices before feeding the child, the main source of drinking water, distance to the main source of drinking water, sharing the toilet with other households, season, intake of (ORS, zinc, and antibiotics), prior hospitalisation, history of vitamin A intake, stunting, distance travelled from home to hospital, duration of diarrhoea, dehydration, watery stool, intravenous rehydration status, duration of hospital stay, and group of children (Rohingya refugees or neighbouring host). aOR and 95% CI values for some variables were missing in **Table 2**, as only the final logistic regression model for each pathogen was presented.

## DISCUSSION

We identified that enteric pathogens were associated with diarrhoea among Rohingya refugees and neighbouring host children in Cox’s Bazar, as well as with demographic factors, care-seeking behaviour, and clinical conditions. We observed higher rotavirus and fewer *Vibrio cholerae* infections among the younger children aged ≤24 months. *Vibrio cholerae* and NTS had a low overall prevalence in both groups, whereas rotavirus was identified overall but was less prevalent during the wet season than the cold season. Lack of handwashing with soap before feeding the child increased the risk of *Aeromonas* infection, whereas intake of oral antibiotics before hospitalisation decreased the risk. Children with rotavirus diarrhoea more commonly had watery stools and longer hospital stays, whereas children with cholera more frequently had intravenous rehydration. Drinking tube well water and the recent intake of vitamin A appeared to be protective against *Vibrio cholerae,* rotavirus, *Aeromonas*, and NTS infections.

We observed a significant association between watery stool and rotavirus infection. The extremely contagious rotavirus causes damage to the epithelial cell lining of the small intestine after intake of contaminated food and water. This infected and damaged epithelium was unable to absorb nutrients and water, resulting in intestinal leakage and fluid loss through diarrhoea [31]. A study from urban Bangladesh also reported the same association in U5 children between two observation periods [32]. Watery stool was found to be one of the predominant symptoms in Thai, African, and Vietnamese children with rotavirus enteric infection [33–35]. We also found that children infected with rotavirus and *Vibrio Cholerae* had a longer duration of hospital stay than those with no pathogen in the stool. A similar result was found in a study conducted in India [36]. Vomiting and dehydration in both entities lengthen the hospital stay [37].

Organisms for enteric infection transmission typically occur through the faecal-oral route, and contaminated food and water are the main sources of environmental transmission. Bangladesh has a special geographical configuration that dominates the formation of cyclones, floods, droughts, tornadoes, and heavy rainfall. Coastal areas are more affected by disasters related to heavy rainfall. Tube wells are one of the main sources of drinking water in Bangladesh. A study conducted in Bangladesh found that drinking water from tube wells was less likely to be contaminated during flooding events. Those who drink from tube wells become protective against rotavirus during the monsoon season [38]. The use of tube wells provides a protective effect against other diarrhoeal diseases in Bangladesh, such as cholera [39,40] and shigellosis [40], especially in flood-prone areas [41]. Tap water was more likely to get contaminated with *Vibrio cholerae* due to its interconnected pipes [42]. We also observed that *Vibrio cholerae,* rotavirus, *Aeromonas*, and NTS infection were less common in children who used tube well as the main source of drinking water. To ensure the availability of drinking water, approximately 20 000 tube wells were installed in Teknaf by different organisations for Rohingya refugees [43]. The use of tube well water might be related to the protection against enteric infections among U5 children.

We observed a significant positive association between *Aeromonas* infection in children and the parents' lack of handwashing with soap and water before feeding them. Handwashing is a mainstay of preventing most enteric pathogens, including *Aeromonas* [44]. We found that handwashing before feeding a child was associated with a reduced risk of *Aeromonas* infection, but not with cholera or other bacteria. However, an observational study from Bangladesh found no significant association between diarrhoea in children and handwashing with or without soap before feeding a child [45]. A systematic review found a 30% reduction in the risk of diarrhoea in children in low- and middle-income countries by handwashing with soap [46]. Proper handwashing with soap and water before feeding the child is one of the five important times among recommendations to prevent diarrhoea in U5 children [47]. Like other enteric bacterial pathogens, *Aeromonas* is also transmitted through contaminated food and water. However, *Aeromonas* can persist in different environmental areas and has a close association with humans and animals. It is present in both aquatic freshwater and marine ecosystems, seafood, meat, soil, and vegetables [15]. *Aeromonas* could survive in different environmental conditions, increasing the risk of transmission and infection with contaminated hands during food preparation or feeding. *Aeromonas* is a ubiquitous bacterium; its survival and mode of transmission urge the necessity of hand hygiene practices to reduce infection [49]. We underscore the necessity of handwashing practices to prevent diarrhoea with enteric pathogens, like *Aeromonas.* Many organisations have carried out extensive campaigns to promote hand hygiene. One is the Bangladesh Rural Advancement Committee, installing thousands of handwashing stations in the refugee camps covering over a million Rohingya population. However, the usage of these stations is still low. Noteworthy factors behind this were people’s inertia, cultural practices, lack of education, and understanding of consequences [6].

We also found that seasonal variation influenced diarrhoeal pathogens in U5 children. During the wet season, there was an increased risk of cholera and NTS infection, whereas the risk of rotavirus infection decreased. Our findings were similar to a prior study which showed the relation between enteric pathogens and seasonal patterns [28]. However, we found no association between seasonal variation and *Aeromonas* enteric infection. A study on the Rohingya population found that enteric bacterial pathogens were more prevalent in the wet season compared to the dry season among U5 children. The opposite result was observed for viral pathogens [27]. Islam and colleagues found a higher *Vibrio cholerae* isolation during the months of April-June/September-November in the Rohingya refugees and neighbouring host populations [2]. A nationwide surveillance study by Khan and colleagues in Bangladesh also found a higher number of cholera cases during the months of March-June (pre-monsoon) and September-October (post-monsoon). [14]. The biannual cholera peak was found in two prior studies [2,14], whereas we found an association between cholera in children and the wet season (July–October) only. In our study, the majority of children were <2 years of age, and cholera was found to be less prevalent in this age group [14]. The heterogeneity of age among the study children might be one of the reasons for the lack of any association between cholera and the hot season (March–June). Moreover, younger children were more prone to enteric infection with rotavirus, which increases during the cold season [37] and decreases during the wet season (July–October) [28].

We found that enteric infection with NTS was more prevalent during both hot and wet seasons. A similar observation was made in a prior study that found that isolation of NTS from diarrhoeal patients showed a rising trend in March and declined after October, peaking in July and August [50]. The seasonal variation of diarrhoeal pathogens might be associated with precipitation, ambient temperature, WASH practices, and occupational behaviour. During the rainy season, heavy rainfall might cause an overflow of faecal matter from unsafe latrines and cause pollution of the food, water sources, residences, and surfaces [51].

We found supplementation with vitamin A, six months prior to the onset of diarrhoea, to have a protective role against diarrhoeal pathogens except for rotavirus. A similar finding was reported in a study in rural Haiti, where an inverse association was observed between diarrhoea and intake of vitamin A in U5 children [52]. Vitamin A has an anti-infectious role by regulating cell growth and differentiation of the immune system [53]. Different studies have shown reduced diarrhoea severity and mortality by vitamin A. A systematic review found a 30% reduction in diarrhoea-specific mortality with vitamin A supplementation in U5 children [53]. There was a 12% reduction in child mortality due to diarrhoea by vitamin A supplementation reported by the United Nations Children's Fund and a meta-analyses [54,55]. However, a meta-analysis found that vitamin A supplementation reduced mortality from diarrhoea, though no continual protective impact on diarrhoea incidence [56]. Vitamin A regulates both the innate and adaptive immune response. Pathogen load and pathogen-specific clinical symptoms are influenced by a definite mechanism of innate and adaptive gastrointestinal immune response. [57]. It was shown that the severity of pathogen-induced diarrhoea and diarrhoea-related infant mortality was reduced by vitamin A supplementation. However, the reduction of faecal cytokine (*i.e.* IL-4, IL-6, IFN-γ) by vitamin A supplementation depends on the type of pathogens and the onset of the pathology [58]. Vitamin A supports the integrity of the gut epithelium by regulating the expression of tight junction proteins that are necessary for gut barrier function and thereby reducing the effects of severe diarrhoea [59,60].

We found that children with NTS infection had a shorter duration of diarrhoea when they arrived at the DTCs. There was a reverse observation from the sub-Saharan Africa site of the Global Enteric Multicenter Study, the longer duration of diarrhoea was an important clinical feature among NTS-positive children [61]. However, in the South Asia site, there was no association between the duration of diarrhoea and NTS isolation [61]. The longer duration of diarrhoea with NTS in Africa was probably due to the high prevalence of human immunodeficiency virus [62]. Several countries, including Bangladesh, identified NTS as a cause of acute diarrhoea [50,63–65].

Similar to previous studies, we observed that young children are more vulnerable to viral gastroenteritis compared to bacterial infection [66,67]. Although we focused on pathogen prevalence, probably immunological factors provide plausible explanations for the predominance of viral infection among young children [68,69].

### Limitations

Our study has some limitations. We used data from DTCs of icddr,b only and could not evaluate the situation of other community health clinics. We did not include children who visited other healthcare facilities or remained at home. We only considered the hospitalised children for analysis, which limits the generalizability of our findings. Usually, children with mild diarrhoea did not visit or were not admitted to the hospital. However, a community-based surveillance study conducted in the Rohingya refugee camp found that bacterial pathogens were present in 42% of stool samples, and viral pathogens were present in 70% of stool samples [27]. A multisite, multi-country birth cohort community surveillance study also detected enteric pathogens both in diarrhoeal and non-diarrhoeal stool of 0–24-month-old children [11]. Some unmeasured confounding factors (*e.g.* food habits and type of foods) could have influenced our outcome and led to potential misinterpretation of the findings. We had no data on breastfeeding duration and prior medical conditions. Therefore, we could not evaluate their influence on the susceptibility of different enteric pathogens. We could not explore airborne transmission of enteric pathogens in an overcrowded setting due to a lack of air pollution data.

## CONCLUSIONS

We highlight notable occurrences of rotavirus infection, whereas the prevalence of cholera, NTS, and Aeromonas infection was low among children under 24 months old. We observed a higher incidence of bacterial diarrhoea in the wet season compared to the dry season. We found the use of Vitamin A supplementation and tube well water to be protective factors for all types of diarrhoea. Pathogen-specific diarrhoeal disease surveillance may serve as an early warning system of impending epidemics and proper resource allocation in vulnerable areas like refugee camps. Based on our findings, we recommend reinforcing the vaccination program, developing WASH infrastructure, and targeted hygiene education for this at-risk population. The major novelty of this study was that we compared the migrants with the local people. Rohingya people are found to be more susceptible to disease due to a lack of preventive intervention. Policy makers may consider taking health initiatives to halt the deterioration of health conditions in such people.

## Additional material


Online Supplementary Document
