# Supplementary material for: Pathogen-specific predicting factors of childhood diarrhoea and their seasonality: evaluation from Rohingya refugees and host population in Cox’s Bazar, Bangladesh
Source: J Glob Health. 2026 Jan 23;16:04024. doi: 10.7189/jogh.16.04024 (PMC12828441; doi:10.7189/jogh.16.04024)
Supplement: Online Supplementary Document [file jogh-16-04024-s001.pdf]

Supplement to: Shaly NJ, Nuzhat S, Sarmin M, Hossain N, Mariam N, Bhuiyan SET, Nabin MAA, Tariqujjaman M, Haque MA, Ahmed D, Faruque ASG, Ahmed T, Chisti MJ. Pathogen-specific predicting factors of childhood diarrhea and their seasonality: evaluation from Rohingya refugees and host population in Cox's Bazar, Bangladesh. J Glob Health. 2026;16:04024.

Table S1. Description of explanatory variables

Sl.	Characteristics	Explanatory variables
1	Scio-demographic characteristics	Age, sex (male or female), study children (Rohingya refugees and neighboring host children), parental educational status (literate or illiterate), number of under-five children in the household, and construction materials of the house (cemented/ non-cemented floor)
2	Breastfeeding status	Study children were categorized based on whether the study children were breastfed or not at the time of enrollment in the surveillance
3	Water, sanitation, and hygiene (WASH) practices	The main source of drinking water (tube well or public tap and others), sanitation (used water seal toilet or not), hand washing practiced with soap and water (before feeding the child and after cleaning the child), treatment of drinking water, and outside food consumption 72 hours before the onset of diarrhea
4	Healthcare-seeking behavior	Intake of antibiotics, ORS, and zinc at home before hospitalization, care seeking from the pharmacy, distance and time to reach DTCs from home
5	Vaccination status	Cholera vaccine, measles vaccine, and vitamin A
6	Nutritional status	Anthropometric measurements (weight, height/length) were measured at the time of enrollment for each child, and WAZ, WHZ, and HAZ scores were calculated. Underweight, wasting, and stunting were considered if WAZ, WHZ, and HAZ scores were <-2, respectively
7	Seasonal variation	The year was categorized into three seasons (cold season: November-February, hot season: March-June, wet season: July- October)
8	Clinical characteristics	Evaluated by duration of diarrhea on admission, presence of abdominal pain, characteristics of the stool (watery or not), dehydration status (presence or absence of dehydration), requirement of intravenous fluid for correction of dehydration, and duration of hospitalization.

Table S2. Sociodemographic and clinical characteristics, care-seeking behavior, and nutritional status in under-five children among Rohingya refugees and neighboring host population attending a diarrhea treatment center in Cox's Bazar, Bangladesh

Variables		U5 Rohingya refugee children n=1479 (%)	U5 host children n=2055 (%)	P value
**Socio-demographic characteristics**				
Female, Sex		591 (39.9)	853 (41.5)	0.355
The median age in months		11.08 (8.16, 15.1)	12.14 (9.08, 17.07)	<0.001
Currently breastfeed		1266 (85.6)	1779 (86.6)	0.410
Monthly family income <9K		1,207 (81.6)	466 (22.7)	<0.001
Illiterate Parent		1279 (86.5)	1626 (79.1)	<0.001
Source of main drinking water	Public tap	1157 (78.2)	48 (2.3)	<0.001
	Tube well	286 (19.3)	1835 (89.3)	
	Others	36 (2.4)	172 (8.4)	
Treatment of drinking water		372 (25.1)	188 (9.1)	<0.001
Used toilet without water seal		1397 (94.5)	1663 (80.9)	<0.001

Shared toilet with other households	1,436 (97.1)	391 (19.0)	<0.001
Practiced hand washing with soap and water	99 (6.7)	214 (10.4)	<0.001
Practiced hand washing with soap before feeding the child	1,344 (92.3)	1,593 (79.2)	<0.001
Practiced hand washing with soap after cleaning the child	1,450 (98.8)	1,969 (96.1)	<0.001
Consumed outside food 72 hours before diarrhea	74 (5.0%)	217 (10.6)	<0.001
Floor material of the house (cemented/ceramic tiles)	470 (31.8)	885 (43.0)	<0.001
Number of people live in the household (median, IQR)	5 (4,7)	5 (4,7)	0.208
Number of Under-five children in the household (median, IQR)	2 (1,2)	1 (1,2)	<0.001
Distance to the main source of water [in feet (median, IQR)]	40 (20,70)	20 (10,40)	<0.001
Got cholera vaccine	243 (16.4)	188 (9.1)	<0.001
Got measles vaccine 1st dose	831/1019 (81.5)	1381/1563 (88.4)	<0.001
Season: Wet season	618 (41.8)	419 (20.4)	<0.001
Cold season	634 (42.9)	1364 (66.4)	
Hot season	227 (15.3)	272 (13.2)	
**Care-seeking behavior**			
ORS intake before hospitalization	1201 (81.2)	1602 (77.9)	0.019
Took zinc before hospitalization	199 (13.5)	398 (19.4)	<0.001
Oral antibiotic before hospitalization	265 (17.9)	811 (39.5)	<0.001
Received vitamin A within last 6 months (48)	577/1349 (42.8)	991/1907 (51.9)	<0.001
Seek healthcare from the pharmacy	264 (17.8)	1667 (81.1)	<0.001
Distance traveled from home to hospital [in Kilometer (median, IQR)]	2.0 (1.0,3.0)	5.0 (3.0, 8.0)	<0.001
**Nutritional status**			
Underweight (WAZ <-2)	472/1476 (31.9)	453/2053 (22.1)	<0.001
Wasting (WLZ <-2)	388/1470 (26.4)	435/2042 (21.3)	<0.001
Stunting (LAZ <-2)	300/1471 (20.4)	327/2046 (15.9)	0.001
**Clinical characteristics**			
Duration of diarrhea >1 day on admission	887 (59.9)	1315 (63.9)	0.015
Presence of abdominal pain	289/1478 (19.5)	637 (31.0)	<0.001
Watery stool	1187 (80.3)	1665 (81.0)	0.570
Dehydration	297 (20.1)	409 (19.9)	0.896
Required intravenous rehydration	71 (4.8)	46 (2.2)	<0.001
Duration of hospital stay [in hours (median, IQR)]	5 (4,12)	8 (5,21)	<0.001

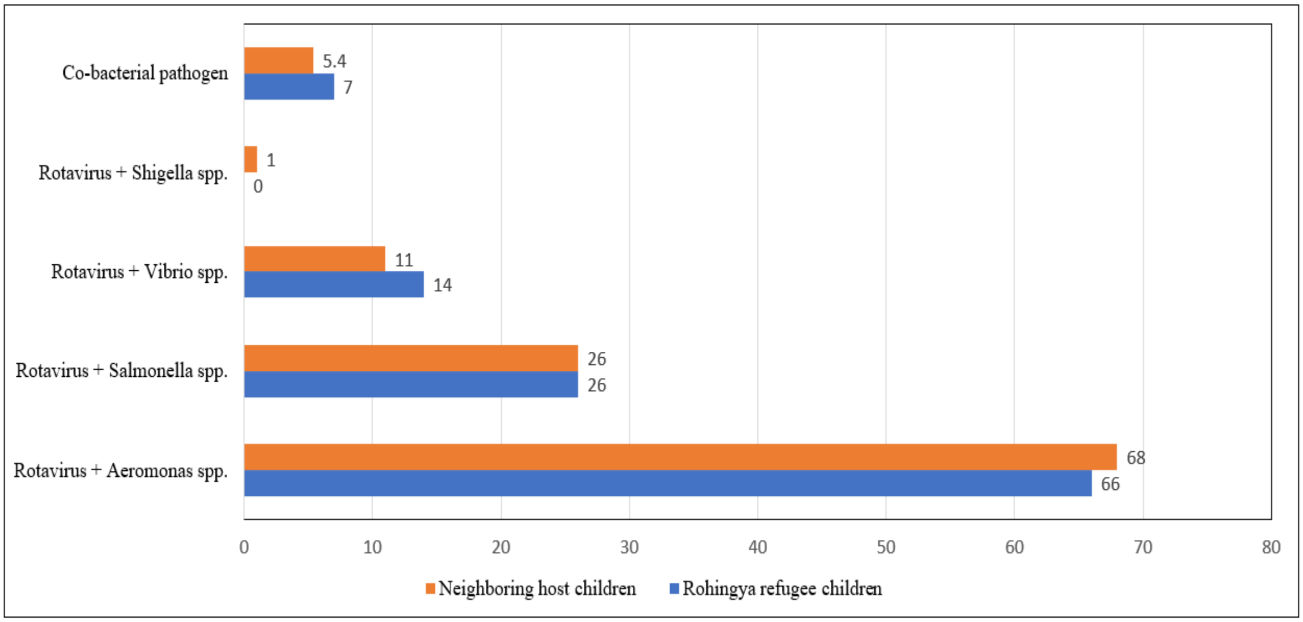

Figure S1. Distribution of mixed pathogens in numbers among the study children

Table S3. Categories of food consumed by study children

	Variables	Types of food
1	Milk-base food	Breast milk, Child formula, milk powder other than milk, cheese or curd, or other food made of milk
2	Protein-base food	Meat, poultry, fish, snails, mollusks, organ liver, egg, beans, nuts, and barbecue, betel nuts (pulse), dal
3	Vitamin-rich fruits and Vegetables	Vitamin A-enriched fruits and vegetables, other fruits, and vegetables

Table S4. Dietary habits of under-five children with *Vibrio cholerae*, Rotavirus, *Aeromonas* spp., and non-typhoidal *Salmonella* (NTS) enteric pathogens among Rohingya refugees and neighboring host population attending the DTCs in Cox's Bazar, Bangladesh

Variables	***Vibrio cholerae*** **n= 83 (%)**	**Rotavirus n= 1278 (%)**	***Aeromonas*** **n= 168 (%)**	**NTS n= 46 (%)**	**No Pathogen n= 1723 (%)**	**P value**
**Dietary Habits of the Study Children**						
Intake of milk-based food (breastfeeding, formula milk, and others)	5 (6.0)	186 (14.5)	24 (14.3)	7 (15.2)	290 (16.8)	0.059
Intake of protein-based food (Fish, meat, beans, and others)	9 (10.8)	104 (8.1)	15 (8.9)	4 (8.7)	155 (9.0)	0.880
Intake of vitamin-rich fruits and Vegetables	10 (12.1)	135 (10.6)	15 (8.9)	4 (8.7)	204 (11.8)	0.653

Table S5. Sensitivity analysis of multiple logistic regression analysis for factors associated with mixed pathogens enteric infection among Rohingya refugees and neighboring host under-five children

**Factors**		**Mixed pathogens aOR (95%CI)**	**P value**
Age groups in months			
	24-59 months	Reference	
	0-11 months	1.14 (0.69, 1.87)	0.608
	12-23 months	0.97 (0.58, 1.62)	0.914
Sex			
	Male	Reference	
	Female	0.80 (0.58, 1.09)	0.156
Intake of composite food, Kunda			
	No	Reference	
	Yes	0.69 (0.50, 0.94)	0.020
The main source of drinking water			
	Public tap	Reference	
	Tube well	0.46 (0.33, 0.65)	<0.001
	Others	0.84 (0.48, 1.49)	0.557
Season			
	Cold season	Reference	
	Wet	1.34(0.96, 1.87)	0.089
	Hot	0.42 (0.24, 0.72)	0.002
ORS intake before hospitalization			
	No	Reference	
	Yes	1.72 (1.11, 2.67)	0.016
Oral antibiotic before hospitalization			
	No	Reference	
	Yes	0 .71 (0.50, 1.01)	0.060
Watery stool			
	No	Reference	
	Yes	1.57 (1.04, 2.37)	0.031
Duration of hospital stay (day)		1.01 (1.00, 1.03)	0.035
Hosmer-Lemeshow p-value			0.5431
